# Bioinformatic analysis of xenobiotic reactive metabolite target proteins and their interacting partners

**DOI:** 10.1186/1472-6769-9-5

**Published:** 2009-06-12

**Authors:** Jianwen Fang, Yakov M Koen, Robert P Hanzlik

**Affiliations:** 1 Applied Bioinformatics Laboratory, University of Kansas, Lawrence, KS 66045, USA; 2 Department of Medicinal Chemistry, University of Kansas, Lawrence, KS 66045, USA

## Abstract

**Background:**

Protein covalent binding by reactive metabolites of drugs, chemicals and natural products can lead to acute cytotoxicity. Recent rapid progress in reactive metabolite target protein identification has shown that adduction is surprisingly selective and inspired the hope that analysis of target proteins might reveal protein factors that differentiate target- vs. non-target proteins and illuminate mechanisms connecting covalent binding to cytotoxicity.

**Results:**

Sorting 171 known reactive metabolite target proteins revealed a number of GO categories and KEGG pathways to be significantly enriched in targets, but in most cases the classes were too large, and the "percent coverage" too small, to allow meaningful conclusions about mechanisms of toxicity. However, a similar analysis of the directlyinteracting partners of 28 common targets of multiple reactive metabolites revealed highly significant enrichments in terms likely to be highly relevant to cytotoxicity (e.g., MAP kinase pathways, apoptosis, response to unfolded protein). Machine learning was used to rank the contribution of 211 computed protein features to determining protein susceptibility to adduction. Protein lysine (but not cysteine) content and protein instability index (i.e., rate of turnover in vivo) were among the features most important to determining susceptibility.

**Conclusion:**

As yet there is no good explanation for why some low-abundance proteins become heavily adducted while some abundant proteins become only lightly adducted in vivo. Analyzing the directly interacting partners of target proteins appears to yield greater insight into mechanisms of toxicity than analyzing target proteins per se. The insights provided can readily be formulated as hypotheses to test in future experimental studies.

## Background

More than half a century has passed since the discovery that reactive electrophilic metabolites derived from xenobiotic agents covalently modify endogenous cellular proteins [1,2]. Since then such covalent binding by reactive metabolites has been strongly correlated with, and is widely believed to be responsible for, the acute organ-damaging effects of a wide range of xenobiotic agents including drugs and natural products [3-5]. Tissue injury is a complex phenomenon. Most tissues are comprised of more than one cell type, and macroscopic tissue injury often involves various secreted chemical mediators such as cytokines, tumor necrosis factor, nitric oxide and pro-inflammatory cells such as leukocytes and macrophages or Kupffer cells. Thus it is important to note that the same compounds that can cause organ damage in vivo can also cause acute cytotoxicity, correlated to protein covalent binding, in isolated metabolically-competent cells in vitro.

It has generally been presumed that protein adduction impairs protein function, leading to disruption of metabolic or signaling pathways, organelle failure, loss of cellular homeostasis, aberrant cell-cell interactions, macroscopic tissue damage through necrosis and/or apoptosis, and in the extreme, organ failure and death. We know that some compounds that give rise to covalently bound residues do not cause toxicity, i.e., *none *of their adducts trigger events leading to cytotoxicity. Likewise some, perhaps many, of the adducts of toxic compounds are ineffective at causing toxicity, but some of the adducts of toxic compounds do trigger events leading to cytotoxicity (and eventually tissue damage and organ injury in vivo). The challenge is to identify which adduct structure(s) on which amino acid residue(s) of which protein(s) are important for toxicity, and the mechanism(s) by which their appearance triggers toxicity while others do not.

Despite extensive investigation, the mechanisms by which covalent binding events trigger cytotoxic outcomes remain largely unclear [6,7]. A major reason for this gap is that only recently has it become technically feasible to identify numbers of individual proteins targeted by xenobiotic reactive metabolites. Early target protein identifications were based on isolating individual adduct-bearing proteins, one at a time, using traditional protein separation methods. By 1997, only 28 proteins targeted by xenobiotic reactive metabolites had been isolated and identified, largely by N-terminal sequencing [6]. In 1998, however, the coupling of 2D gel electrophoresis with mass spectrometric methods of protein identification literally revolutionized the field [8]. Since then both the number of known target proteins and the number of small-molecule adduct-forming xenobiotics studied have increased nearly ten-fold. To help keep track of this information and to facilitate its analysis, we recently built the Reactive Metabolite Target Protein Database (TPDB) [6,9,10]. All proteins listed in this database came from studies using live animals or intact living cells.

As the number of known target proteins grew, so did the hope of elucidating mechanistic pathways connecting covalent adduction events to the observed cytotoxic outcomes. For example, we and others [7,11-14] have attempted to make sense of lists of target proteins by arbitrarily grouping them into categories according to function, but this approach has done little to reveal to a unifying mechanism of toxicity caused by a variety of reactive metabolites [6,7]. Another way to analyze target proteins is to sort them into Gene Ontology (GO) categories [15] and determine whether any show an over-abundance of target proteins relative to statistical expectations. Similarly, KEGG biological pathway analysis [16] can identify metabolic pathways in which target proteins are over-represented. Such analyses could potentially indicate a functional connection between target protein adduction and biological consequence in the context of systems biology [12].

In living cells, proteins interact extensively with other proteins, forming protein-protein interaction (PPI) networks that sense and respond to the abundance or status of other network proteins. Since endogenous post-translational modifications of proteins are well-known to perturb PPI networks comprising intracellular signaling cascades [17-20], it is plausible to hypothesize that protein modification through adduction by xenobiotic reactive metabolites could constitute an aberrant form of signaling leading to cytotoxic consequences. Thus, inspection of the interacting partners of reactive metabolite target proteins might shed new light on the path from protein adduction to cytotoxicity.

In addition to the question of how protein adduction leads to cytotoxicity, it is also of interest to know what features of a protein, beyond simple abundance, determine whether or not it is likely to be a target for reactive metabolites. Electrophilic xenobiotic metabolites can be classified broadly as having acylating or alkylating activity [6,10]. The former tend to attack lysine side chains, while the latter tend to attack predominantly cysteine, histidine and lysine side chains. Despite the commonplace occurrence of these side chains in most proteins, it is well established that protein adduction in living cells is remarkably selective, with some abundant proteins experiencing little adduction while some low-abundance proteins experience high levels of adduction [7,13]. Nevertheless, the protein features that determine susceptibility to adduction are almost completely unknown [21]. Since many protein features can now be calculated or predicted using software programs, the analysis of target proteins using feature selection algorithms could potentially shed light on this important question [22]. Feature selection algorithms operate differently from conventional statistical (correlative) studies of individual features considered independently from each other. Such algorithms can identify which features among many contribute the most to determining a complex behavior such as relative susceptibility to adduction by electrophiles.

In this paper we report our efforts to use bioinformatics approaches to elucidate the interactions between reactive metabolites, their cellular target proteins, and the other proteins that interact directly with the target proteins. In brief, we first analyzed 171 proteins targeted by reactive metabolites from one or more of 18 different protoxins and found that a number of GO categories and KEGG pathways were significantly enriched with some of these target proteins. We then selected 28 proteins known to be adducted by reactive metabolites of at least 3 different protoxins and found that 21 of them had a total of 165 directly interacting partners. GO and KEGG pathway analysis of the combined 186 proteins revealed several categories to be highly significantly enriched by target proteins and/or their directly interacting partner proteins. Finally, we applied machine learning methods to analyze the properties of 62 rat liver proteins targeted by reactive metabolites of thiobenzamide and 45 rat liver proteins targeted by reactive metabolites of bromobenzene, in an effort to identify properties that help to distinguish whether a protein is likely to be a target of reactive metabolites.

## Methods

The identities of the target proteins used in this study, and the structures and names of the protoxins whose metabolites bind to them, are freely available from the reactive metabolite target protein database [6,9,10]. For the Gene Ontology and KEGG Pathway analyses we used all 171 proteins listed in the TPDB as of February 2008. For the protein-protein interaction analysis we used the "rank by number of hits" function within the TPDB to select the 28 proteins most commonly targeted by different reactive metabolites. For the machine learning study we used 37 proteins targeted by thiobenzamide metabolites, 20 proteins targeted by bromobenzene metabolites, and 25 proteins targeted by both (82 proteins total). To create a negative learning dataset, all rat proteins in UniProtKB [23] were downloaded on May 5, 2008. A software program CD-HIT [24] was used to filter redundant sequences in the dataset at the level of 80% identity. We also eliminated all sequences with lengths less than 40 residues and subtracted all known target proteins to arrive at a set of 11482 proteins in the negative (non-target) learning dataset. Although the list of target proteins is incomplete, the percentage of all proteins in living cells that actually become detectably adducted by reactive metabolites is relatively small (< 10% based on comparisons of 2D gels vs. their autoradiograms; [7,13]). Thus the vast majority of proteins in the negative dataset are considered as non-target proteins. In addition, the Random Forest algorithm used to analyze target vs. non-target proteins (see below) is relatively tolerant of small amounts of "noise" in the data.

### Software programs and databases used in the study

The Gene Ontology project [15] is a collaborative effort to develop standard vocabularies (ontologies) that describe gene products in terms of their associated biological processes, cellular components and molecular functions in a species-independent manner. The controlled vocabularies are hierarchically structured (multiple parent levels are permitted) so that they can be queried at different levels. KEGG [16] is a widely used database of biological pathways. The January 2008 version of the Human Protein Reference Database (HPRD) was downloaded from the HPRD website [25]. The binary protein-protein interactions (8919 proteins in 34364 distinct PPIs) were then imported into Cytoscape, an open source bioinformatics software platform for visualizing and analyzing biological interaction networks (http://www.cytoscape.org/). The Cytoscape plugin BiNGO was used to determine and visualize statistically overpopulated GO categories in a set of genes [26].

### Random forest models and feature extraction

We used a random forest package [27] implemented in the "R" environment [28] for this study. Since random forest models are usually insensitive to the model parameters, we used the default parameters. Values for a set of 211 protein features were calculated using various software programs or in-house scripts (Table 1). These features include protein composition in terms of amino acid residues, predicted secondary structures, solvent accessibility, and others. Although the predictive model might have benefited from incorporating elements of protein three-dimensional structure, such information is not available for the vast majority of target proteins. Therefore we used only sequence information and predicted secondary structures. Since the sequence of a protein is a major determinant of its structure, it is expected that features calculated from sequence information alone may be sufficient to distinguish target proteins from other proteins. Software programs for predicting a broad spectrum of other protein properties such as secondary structure, solvent exposure, instability index and others are mature and have found many applications [29,30].

**Table 1:** Sequence parameters

**Protein feature**	**Number of features**	**Calculated with**	**Remarks and references**
Amino acid residue composition	40	In-house script	Numbers and percentages of 20 amino acid residues
Numbers and percentages of positive residues, negative residues, all charged residues, net charges	8	In-house script	
Sequence length	1		
Predicted isoelectric point	1	ProtParam	[37]
Number of C/H/N/O/S atoms	5	ProtParam	[37]
Instability index	1	ProtParam	Predicted based on dipeptide composition [37]
Instability class	1	ProtParam	Proteins with predicted in vivo half life ≥ 40 hrs considered as stable [37]
Aliphatic index	1	ProtParam	[37]
Gravy hydropathy index	1	ProtParam	[37]
Predicted percentage of sheet, helix and coil	3	psipred	[40]
Predicted percentage of buried/exposed residues	2	Accpro	[47]
Predicted disordered regions including length of the longest coil, length of all coils, percentage of the longest coil, percentage of all coils; and corresponding features for rem465 and hotloop	12	disembl	[24]
Tripeptide features (5 * 5 * 5)	125	In-house script	

The dipeptide or tripeptide composition of proteins has been found to correlate to a number of protein properties. For example, Guruprasad *et al. *found a correlation between the dipeptide composition and the stability of proteins [31]. More recently, tripeptide composition was applied in a machine learning model to predict protein-protein interactions [29]. In this study, the 20 amino acids were sorted into just five groups according to their physicochemical properties: hydrophobic, GAVLIMPFW; polar, YTSNQ; positive, RKH; negative, DE; the fifth group contained only cysteine because of its unique ability to form disulfide bonds. Therefore the number of possible tripeptide features according to this classification scheme is 5*5*5 or 125.

### Ranking feature importance

The random forest approach offers several methods to assess the importance of features based on their contributions to the correctness of the resulting classification. These methods include i) the mean decrease in accuracy for each class, ii) the mean decrease in accuracy over all classes, and iii) the mean decrease in the Gini impurity criterion [32]. In this study we used the mean decease in accuracy for the minor class (target proteins) because in previous work we found it to give more accurate results than other measures for ranking feature importance [22].

### Performance evaluation

For imbalanced data (i.e., comparisons of small vs. large data sets), the overall classification accuracy is not an appropriate measure of performance because very high accuracy can be achieved simply by predicting every case as the majority class (i.e. the control proteins) as opposed to predicting the minority class (i.e. target proteins). In this study, we used three indicators of performance: sensitivity, specificity, and area under the curve (AUC) for the receiver operating characteristic (ROC) curve. We ranked all cases in the dataset according to the predicted likelihood of positive status and then employed that rank order to identify correctly classified true positives. We then used these results to generate an ROC curve (i.e., a plot of the true positive rate (sensitivity) against the false positive rate (1 – specificity)). The area under the ROC curve represents the trade-off between sensitivity and specificity over the whole range of data. An AUC of 1 represents a perfect prediction model while an AUC ≥ 0.9 is considered excellent, an AUC between 0.8 and 0.9 is considered good, and an AUC in the range of 0.7–0.8 is fair.

## Results and discussion

### Bioinformatic analysis of known target proteins

To identify GO terms significantly enriched with target proteins we used BiNGO [33], a plugin of the Cytoscape software suite [34], to map target proteins to GO categories. BiNGO uses a hypergeometric test to search for predominant categories in terms of p-values and GO diagrams. For this experiment we used all 171 target proteins in the TPDB (as of January 2008) and found that 163, 159 and 145 of them, respectively, were represented (by their genes) in the Molecular Function, Biological Process, and Cellular Component categories of the Gene Ontology classification system (Tables 2 and Additional files 3 and 4). A complete listing of terms having a false discovery rate (FDR) < 1.0E-03 is presented in Additional file 5, while graphical representations of enriched GO categories and their hierarchical structures are presented in Additional files 1 and 2.  

**Table 2:** Representative Gene Ontology terms significantly enriched in target proteins.^a^

**GO-ID**	Category Description	**Corrected *p*-value**	**Number of target proteins selected**	**Total number of proteins in category**	**Fraction of population as targets**
	***Molecular Function***				
3824	catalytic activity	1.45E-19	115	5085	0.023
16491	oxidoreductase activity	2.76E-12	40	916	0.044
16209	antioxidant activity	2.93E-09	11	57	0.193
51920	peroxiredoxin activity	9.34E-08	5	6	0.833
16684	oxidoreductase activity, acting on peroxide as acceptor	5.71E-07	8	40	0.20
4364	glutathione transferase activity	3.30E-06	7	34	0.206
9031	thioredoxin peroxidase activity	3.30E-06	4	5	0.8
51082	unfolded protein binding	9.02E-05	9	115	0.078
5504	fatty acid binding	9.42E-04	5	37	0.135
	***Biological Process***				
19752	carboxylic acid metabolic process	1.10E-14	35	524	0.067
6519	amino acid and derivative metabolic process	1.02E-08	23	362	0.064
9308	amine metabolic process	1.53E-08	24	414	0.058
6950	response to stress	4.82E-08	36	978	0.0369
6805	xenobiotic metabolic process	2.31E-07	8	32	0.25
9410	response to xenobiotic stimulus	2.74E-07	8	33	0.242
6979	response to oxidative stress	4.38E-07	12	110	0.109
6457	protein folding	1.28E-06	15	207	0.072
	***Cellular Component***				
5737	cytoplasm	4.59E-26	108	3936	0.027
44444	cytoplasmic part	3.71E-23	91	2969	0.031
5739	mitochondrion	2.31E-16	41	750	0.055
5788	endoplasmic reticulum lumen	4.95E-10	8	20	0.4
5829	cytosol	2.95E-09	25	494	0.051
5783	endoplasmic reticulum	9.68E-09	25	525	0.048
5793	ER-Golgi intermediate compartment	2.07E-06	6	23	0.261
5625	soluble fraction	2.56E-06	15	260	0.058

^a ^GO analysis was performed using BinGO in Cytoscape. The rat proteome was used as the background for comparison. A complete listing of terms having a false discovery rate (FDR) < 1.0E-03 is presented in Table S3 (see Additional file 5). Also available in the Additional files are graphic representations of enriched GO categories and their hierarchical structures (Figures S1 and S2 in Additional files 1 and 2, respectively).

Of the 171 known targets, 115 sort into the broad Molecular Function subcategory of catalytic activity, while only 11 sort into the much more specifically-defined subcategory of antioxidant activity. Nevertheless, both results are highly significant statistically. Likewise, 125 of the 171 target proteins sort into the broad Biological Process subcategory of "metabolic process" while only 8 sort into the subcategory of "xenobiotic metabolic process." Again, both of these results are highly significant statistically. However, the results of sorting target proteins into various Cellular Component subcategories must be viewed with some caution for several reasons. First, some target protein identification studies analyzed whole tissue samples whereas others analyzed only selected subcellular fractions of tissue homogenates. Second, a majority of the enzymes associated with bioactivation of xenobiotics to reactive metabolites are located in the endoplasmic reticulum (microsomal fraction) of the cell. For certain metabolites (e.g. trifluoroacetyl chloride formed by P450-catalyzed oxidation of halothane, CF_3_CHBrCl), the reactivity of the metabolite may limit its ability to diffuse from the site of formation to other sites within the cell.

To map target proteins to KEGG pathways we used DAVID Bioinformatics Resources, an online database for annotation, visualization and integrated discovery developed by NIAID NIH [35]. We found that of the 171 known target proteins, 101 are associated with one or more KEGG pathways, 15 of which are specifically enriched (p ≤ 0.001) compared to statistical expectations (Table 3). Pathways involved in glycolysis, gluconeogenesis and glutathione metabolism pathways are among the most enriched.

**Table 3:** KEGG pathways significantly enriched in target proteins.^a^

Pathway Name	**Corrected *p*-value**	**Number of target proteins selected**	**Total number of proteins in pathway**	**Fraction of population as targets**
Glycolysis/Gluconeogenesis	2.96E-08	12	45	0.267
Glutathione metabolism	1.25E-06	9	30	0.300
Carbon fixation	9.92E-06	7	19	0.368
Pyruvate metabolism	1.25E-05	8	29	0.276
Urea cycle and metabolism of amino groups	1.38E-05	7	20	0.350
Arginine and proline metabolism	5.63E-05	7	25	0.280
Metabolism of xenobiotics by cytochrome P450	7.13E-05	9	50	0.180
Limonene and pinene degradation	2.70E-04	5	12	0.417
Propanoate metabolism	4.02E-04	6	23	0.261
Glutamate metabolism	4.97E-04	6	24	0.250
Phenylalanine metabolism	6.97E-04	5	15	0.333
3-Chloroacrylic acid degradation	7.50E-04	4	7	0.571
Fatty acid metabolism	8.59E-04	7	40	0.175
Nitrogen metabolism	9.09E-04	5	16	0.313
Cysteine metabolism	9.09E-04	5	16	0.313

^a^Pathway mapping was performed using DAVID, and 101 of 171 target proteins could be mapped to one or more KEGG pathways. The subset of rat proteins that are allocable to KEGG pathways includes 3396 proteins.

Analyses such as those presented above are based on the principles of statistics; thus the observed enrichments of target proteins in certain GO categories and KEGG pathways are *a priori *significant, at least in the statistical sense. For example, 115 of the 171 target proteins in the Molecular Function GO category sorted into the catalytic activity subcategory (which contains 5085 genes overall; Table 2). Although this result is highly significant statistically (p = 1.45E-19), these 115 targets represent only 2% of all proteins in this broad subcategory. Thus, regardless of its statistical significance, this finding can at best hint at a biologically significant mechanistic connection to toxicology. In contrast, only 5 of the 171 target proteins sorted into the peroxiredoxin activity subcategory. This result too is highly significant statistically (p = 9.43E-08), but since this entire subcategory contains only 6 proteins, the finding that five of them (83%) are targeted by reactive metabolites suggests that perturbation of this Molecular Function by protein adduction could perhaps be significant biologically. A number of other Molecular Function and Biological Process GO terms that are overpopulated with target proteins have been individually implicated in the context of reactive metabolite toxicity (e.g., peroxidase activity, response to oxidative stress, antioxidant activity, etc.). Likewise 101 of the171 target proteins (59%) were also enriched in certain KEGG pathways (Table 3), some of which (e.g., glycolysis, urea cycle, glutamate metabolism, cysteine metabolism and, not surprisingly, metabolism of xenobiotics by cytochrome P450) have also been linked to potential mechanisms of chemical cytotoxicity.

Collectively, the highlighting of certain GO terms and KEGG pathways via the more independent and more comprehensive bioinformatics approach taken here is intriguing and encouraging. While findings such as this can help to formulate or reinforce hypotheses about mechanisms of reactive metabolite cytotoxicity, each such hypothesis will need more extensive testing before it can be taken seriously as a significant contributor to the overall mechanism of cytotoxicity. In the end, however, it must be conceded that the systematic global analyses of all known target proteins affords only a modest advance toward elucidation of mechanisms beyond that afforded by gazing at lists of arbitrarily-grouped target proteins of single protoxicants [6,7]. To go beyond this limitation, we searched for and analyzed non-target proteins that interact directly with known target proteins, as described below.

### Bioinformatic analysis of common target proteins and their interacting partners

#### Protein-protein interaction data analysis

From the total set of 171 target proteins in the TPDB we selected 28 rat or mouse proteins that are common targets for multiple reactive metabolites (Table 4). Because published PPI data for rat and mouse are quite sparse, whereas extensive PPI data are available for human proteins, we first found the human orthologs of these 28 rat or mouse target proteins by searching against human proteins in the NCBI non-redundant protein database (nr) using the online NCBI BLAST server. The search results were manually inspected and the most significant matches were selected (see Additional file 3). The minimum and average percent identity of the target proteins and their human homologs is 69% and 88%, respectively. The maximum difference in sequence lengths for these protein pairs is only 2.3%. Thus, the selected human proteins are very likely to be orthologs of the target proteins. We then searched for the respective interacting partners of the target protein orthologs in the Human Protein Reference Database [25]; this approach is based on the common assumption that PPIs are conserved across species [36]. We found a total of 165 proteins to be directly interacting partners to 21 of the 28 target protein orthologs. A full list of these interacting proteins is available in the Additional Materials (see Additional file 4).

**Table 4:** Summary of interacting partner analysis for human orthologs of 28 rat or mouse proteins commonly targeted by multiple different reactive metabolites ^a^

**Entrez id**	**Gene symbol**	**Human Protein name**	**Number of interacting partner proteins found**
213	ALB	Albumin	12
217	ALDH2	Aldehyde dehydrogenase 2	7
383	ARG1	Arginase	2
761	CA3	Carbonic anhydrase III	3
2023	ENO1	Enolase 1	6
2168	FABP1	Fatty acid binding protein 1	2
2923	PDIA3	Protein disulfide isomerase A3	11
2944	GSTM1	Glutathione S-transferase Mu-1	4
2946	GSTM2	Glutathione S-transferase Mu-2	4
3309	HSPA5	BIP	31
3312	HSPA8	Heat shock 70 kDa protein 8	43
3417	IDH1	Isocitrate dehydrogenase 1	1
5034	P4HB	Protein disulfide isomerase	15
5037	PEBP1	Raf kinase inhibitor protein	18
5230	PGK1	Phosphoglycerate kinase 1	4
7170	TPM3	Tropomyosin 3	13
7276	TTR	Transthyretin	14
7295	TXN	Thioredoxin	10
8991	SELENBP1	Selenium binding protein 1	4
10130	PDIA6	Protein disulfide isomerase P5	2
10961	ERP29	Endoplasmic reticulum protein 29	4
1109	AKR1C4	Aldo keto reductase family 1, member C4	0
1652	DDT	D-dopachrome tautomerase	0
2052	EPHX1	Epoxide hydrolase	0
2184	FAH	Fumarylacetoacetase	0
2593	GAMT	Guanidinoacetate N-methyltransferase	0
10247	HRSP12	Translational inhibitor protein p14.5	0
51733	UPB1	Beta ureidopropionase	0

^a ^No interacting partners were found for 7 of the 28 proteins. See Additional file 5 for full listing of all the interacting partners found.

#### GO and KEGG pathway analysis of common targets and their interacting partners

We used BiNGO to identify GO categories significantly overpopulated with target proteins or their first interacting partners. Of the 186 target proteins and direct partners analyzed, 182 link to one or more GO terms. As summarized in Table 5, highly significant enrichment was observed in the subcategories of protein folding, unfolded protein binding, response to unfolded protein, and apoptosis. In addition to the statistical significance of the sorting results, the fact that adduction affected a relatively large fraction of the proteins in these categories suggests that these results may also be biologically significant in terms of the degree to which important processes involving these proteins may be impaired or altered by target protein adduction. Of the 186 target proteins plus partners, we found 96 to be involved in one or more KEGG pathways, eight of which are significantly overpopulated compared to statistical expectations (Table 6). Among them, the MAP kinase signaling pathway had the most significant enrichment. This result is particularly intriguing, since this association was *not *found when we analyzed only the reactive metabolite target proteins themselves without including their interacting partners.

**Table 5:** GO categories with an over-representation of target proteins or their interacting partners

**GO-ID**	Category Description	**Corrected *p*-value**	**Number of target proteins selected**	**Total number of proteins in category**	**Fraction of population as targets**
	***Molecular function***				
51082	unfolded protein binding	3.26E-09	14	113	0.124
	***Biological process***				
6457	protein folding	1.65E-17	28	256	0.109
6915	apoptosis	4.63E-09	30	676	0.044
6986	response to unfolded protein	4.63E-09	11	61	0.18
	***Cellular component***				
5783	endoplasmic reticulum	2.83E-06	25	669	0.037

**Table 6:** KEGG pathways containing target proteins or their interacting partners

***Pathway Name***	***Corrected p-value***	**Number of target proteins selected**	**Total number of proteins in pathway**	**Fraction of population as targets**
MAP kinase signaling pathway	4.26E-05	19	259	0.073
Antigen processing and presentation	6.66E-05	10	80	0.125
Alzheimer's disease	1.11E-04	6	28	0.214
Long-term potentiation	5.78E-04	8	65	0.123
Neurodegenerative disorders	0.0011	6	39	0.154
Long-term depression	0.0018	8	75	0.107
Arginine and proline metabolism	0.0018	7	34	0.201
Adipocytokine signaling pathway	0.0054	7	73	0.096

Apparently, looking beyond just target proteins and considering their first interacting partners may provide a deeper look into potential mechanisms of cytotoxicity. The potential indirectness of analyzing human orthologs of rat and mouse target proteins (necessitated by the paucity of information about rat and mouse vs. human PPIs) was offset by focusing on just 28 proteins known to be targeted by multiple different reactive metabolites. Comparing the results of Table 5 to those of Table 2 shows that the GO Molecular Function subcategory "unfolded protein binding" was again highlighted, but this time with much higher statistical significance (3.3E-09 vs. 9.0E-05) and much higher fractional coverage of the class (0.124 vs. 0.078). The significance and coverage of the Biological Process "protein folding" are greatly increased in Table 5 vs. Table 2. In addition, Table 5 also lists two terms having both high statistical significance and high fractional coverage that are absent from Table 2, namely, "apoptosis" and "response to unfolded protein," both of which have high relevance to cytotoxicity.

In terms of KEGG pathway analysis of targets and their partners (Table 6), the flagging of the MAP kinase pathway is particularly noteworthy. This finding is consistent with independent experimental evidence that also strongly supports a role for this pathway in apoptosis and other pathological cellular responses to toxic chemicals [37-40]. There is also considerable interest in the involvement of the immune system in some forms of reactive metabolite-mediated toxicity in vivo [4,5]. The flagging of four pathways related to the nervous system was unexpected, and will require further analysis to evaluate. Nevertheless, it appears that our approach of considering not just target proteins but also the proteins with which they interact in the cell may provide clues to downstream events important to cytotoxicity and thereby help in elucidating mechanisms of cytotoxicity. Stated another way, it is perhaps essential PPIs rather than proteins per se that are the important targets of reactive metabolites in living cells.

### Machine learning approaches to elucidate factors that differentiate target from non-target proteins

In addition to identifying the target proteins whose modification appears to initiate cytotoxic responses, we also examined them more specifically to elucidate protein features that predict susceptibility to reactive metabolites. Because the reactive metabolites of the 18 protoxins included in the TPDB span a wide range of chemical reactivity and hydrophobicity, and because for many of them only a few target proteins are known, we decided to use the set of 82 proteins that are targeted in vivo by thiobenzamide metabolites (n = 62), bromobenzene metabolites (n = 45) or both (n = 25) for this study. Thiobenzamide forms a reactive acylating metabolite with a very strong preference for reaction with amine side chains on lysine [7] or phosphatidyl-ethanolamine (PE) lipids [41]. On the other hand, prior analyses of proteins adducted by bromobenzene metabolites indicates a strong preference for adduction on cysteine sulfhydryls [42,43] as opposed to lysine or histidine [44] or PE lipids [41]. Thus, one might expect the composition of the proteins targeted by these two metabolites to reflect their differing chemical selectivities to at least some extent.

To investigate this we used the random forest algorithm developed by Breiman [32]. Random forest is an ensemble approach that combines many individual classifiers to formulate a robust composite classifier. It is particularly suitable in classifying high-dimensional and noisy data, and it can handle a mixture of both categorical and continuous predictors such as those in Table 1. Each of the classifiers is built on a bootstrap sample of the data and utilizes a random subset of the available variables (predictors) without pruning to obtain low-bias trees. The random forest algorithm has been applied to a broad range of classification tasks and has demonstrated superior performance compared to other classification algorithms [32].

We used five-fold cross validation to estimate the performance of the classification models. In brief, 82 target proteins and the 11482 rat proteins taken as a negative control set (see Methods) were randomly split into 5 equal portions. One portion was reserved as a test data set while the other four were pooled and used as a training set. A random forest classifier with 10,000 trees was built using 211 protein features (see Methods section) and the performance of the model was then evaluated using the reserved dataset. This process was repeated four times, each time starting with a different one of the five subsets of proteins as the test set. The results from all five repeats were then combined to afford an overall performance estimation.

**Figure 1:**
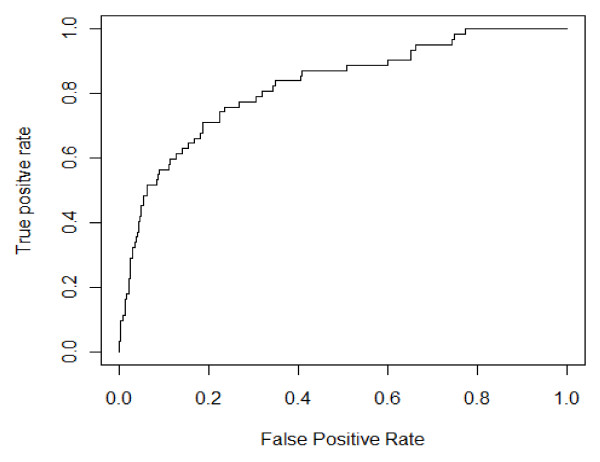
**ROC curve using the false-positive rate and true positive rate to evaluate the performance of the model to predict target proteins**. The area under the curve is 0.857.

**Figure 2:**
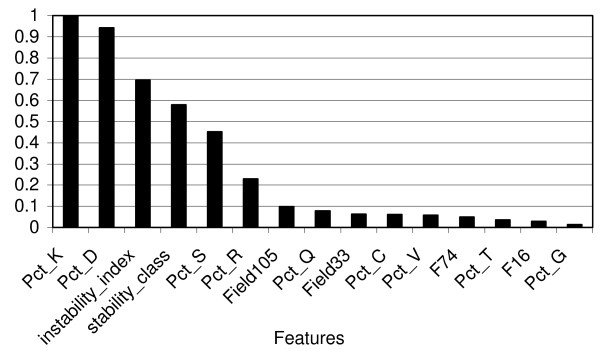
**Relative importance of the top 15 features as ranked by the random forest algorithm**.

For our model the ROC curve in Figure 1 has an AUC of 0.857, while the specificity and sensitivity of the model are 0.710 and 0.784, respectively. Thus this simple model appears to have fairly good predictive power. The relative importance of the 211 protein features used in the model was ranked using the random forest algorithm and the top 15 are displayed in Figure 2. We also estimated the performance of models built using only these top 15 features. The ROC curve of this truncated model has an AUC of 0.779, somewhat inferior to the AUC of 0.857 for the model using all features, suggesting, not surprisingly, that protein targeting by reactive metabolites is a complicated process that depends on many factors.

**Figure 3:**
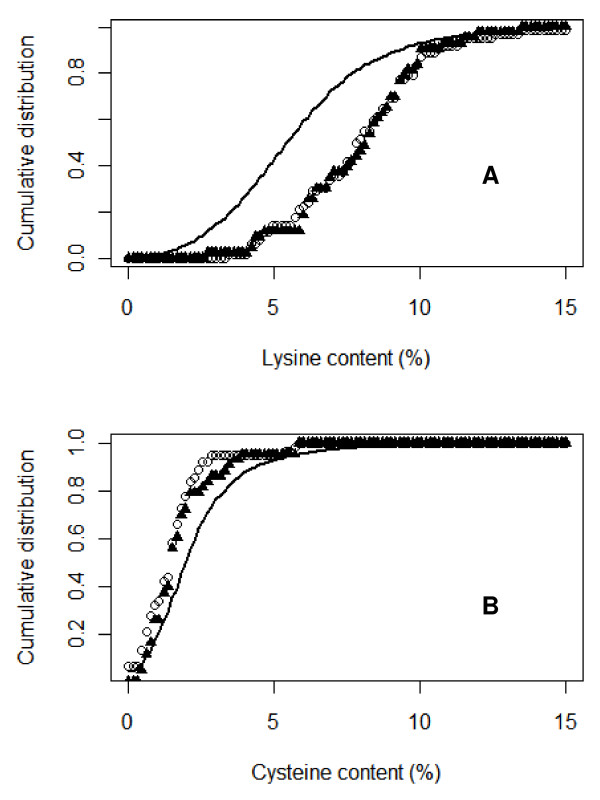
**Cumulative distribution of lysine (panel A) and cysteine (panel B) for 62 thiobenzamide target proteins (open circles), 45 bromobenzene target proteins (filled triangles) and 11482 non-target proteins (solid line)**.

Interestingly, the most decisive feature of the full model is the percentage of lysine in the proteins. This is consistent with the fact that the reactive iminosulfinic acid metabolite of thiobenzamide reacts preferentially with amine groups such as those on lysine side chains. Figure 3A shows a plot of the cumulative distribution of lysine in thiobenzamide target vs. non-target proteins. This plot indicates, as might be expected, that lysine residues are more common among thiobenzamide targets than non-target proteins, but surprisingly, the same is true for bromobenzene target proteins. Figure 3B shows a similar plot for the distribution of cysteine among target vs. non-target proteins. Surprisingly, both sets of target proteins actually show somewhat *lower *cysteine content than the non-target proteins, and again there is no apparent difference between thiobenzamide vs. bromobenzene targets despite the extremely different chemical reactivities of their respective reactive metabolites toward specific protein nucleophiles. Consistent with the results of Figure 3B, Figure 2 also indicates that cysteine content is a relatively unimportant factor for distinguishing target from non-target proteins in vivo, at least for reactive metabolites of bromobenzene and thiobenzamide.

Dennehy et al. investigated the reaction of two model cysteine-reactive electrophiles (IAB, a cytotoxic iodoacetamide derivative, and BMCC, a reactive but non-cytotoxic maleimide derivative) with proteins from the nuclear and cytosolic fractions of HEK293 cells [21]. They found that 89% and 85%, respectively, of 539 proteins that became adducted were selectively adducted at only one or two cysteines per protein. The overlap of target proteins hit by IAB vs. BMCC is only about 20%. In an attempt to explain this selectivity they analyzed the frequency of occurrence of lysine, arginine, histidine or a second cysteine within the first five residues on either side of the adducted cysteine. Some differences in the frequencies were noted for target vs. non-target proteins, but unfortunately, the most likely reasons for the selective reactivity of only a few cysteines among many, namely, protection by intramolecular disulfide formation and/or steric occlusion within the protein, could not adequately be evaluated because such structural information is generally not available for a majority of proteins. In our analysis of features of target proteins we used only two large sets of targets, one from a lysine-selective metabolite and one from a cysteine-selective metabolite. The comparative unimportance of cysteine (vs. lysine) in differentiating target proteins from non-target proteins probably indicates that while a given reactive metabolite may require a specific type of target nucleophile, the target site is only one factor, and a possibly not a sufficient factor overall, for differentiating target from non-target proteins.

The second most important feature of target proteins is the percentage of aspartic acid. Aspartic acid residues are generally not considered to be nucleophilic target sites for most reactive electrophilic metabolites. It is possible that the higher content of aspartic acid provides negative charges to balance the positive charges resulting from a higher percentage of lysine in target proteins; however, the equally negatively charged glutamic acid did not play a significant role in distinguishing target proteins (Figure 2). Moreover, we find that the correlation between the content of lysine vs. aspartic or glutamic acid in the non-target set is insignificant (0.183 and 0.363 respectively), in agreement with the wide range of pI values that proteins span. Clearly more work will be required to understand why some abundant proteins receive little adduction while some low-abundance proteins become more heavily adducted by a given reactive metabolite.

The third most important feature of target proteins is their predicted index of instability (with respect to physiological degradation and turnover). If we consider proteins with a calculated instability index ≥ 40 as unstable, and those with an index < 40 as stable [31], then 57 out of the 82 target proteins (70%) are classified as stable while only 26% of the proteins in the negative learning set (i.e., 2970 out of 11482) are classified as stable (Fisher exact test *p *= 2.26E-16). The relative instability of a covalent metabolite-protein adduct can arise from 1) chemical instability of the covalent linkage, 2) a high intrinsic rate of turnover of the protein itself, or 3) an enhancement of protein turnover induced by adduction. *A priori*, the stability of a protein can influence both the detection and analysis of its adducted forms and the cellular consequences of its adduction (e.g. cytotoxicity). Surveys of target proteins are usually conducted at just a single time after dosing, chosen to maximize the total amount of covalent binding. Proteins whose turnover is rapid relative to the endpoint time will thus experience a lower exposure to reactive metabolite and a greater likelihood of removal by degradation, resulting in a lower apparent rate of adduction compared to proteins that turnover slowly. If adduction flags a protein for accelerated destruction, the result will be the same. This may be one reason that adduct densities vary so widely across a target proteome (for example, see [13]). If adduct density is lowered sufficiently (to the edge of detectability by phosphorinaging, for example), the protein may then be missed altogether as a target. On the other hand, since a relatively high proportion of target proteins turn over less rapidly than non-target proteins, their adduction may result in a more persistent perturbation to endogenous PPIs and signaling mechanisms and may therefore contribute more to cytotoxicity.

Interestingly, Lin et al. [45] recently found a major difference between IAB and BMCC in terms of adduct stability in vivo (as monitored by Western blotting of cell extracts). Both agents form covalent adducts that do not dissociate chemically or in cell-free fractions, but in living cells, the extent of protein adduction by IAB increases continuously over 6 hours, whereas adducts formed by BMCC peak at around 20 minutes after exposure and diminish rapidly thereafter. Their disappearance is strongly retarded at 4°C, suggesting that the disappearance may be enzyme mediated. Two other acetamide/maleimide pairs showed similar differences in protein adduct stability. Lin et al. noted that the rapid clearance of maleimide adducts coincided with their relative lack of toxicity and suggested that protein adduct stability is a critical requirement for the induction of cellular responses. Their observation of declining adduct levels over time can be explained by reversal of the adduction process, and/or by the degradation of protein molecules bearing adducts. Further work will clearly be required to address these possibilities, but it is encouraging that results of both bioinformatic and laboratory analysis of target proteins are pointing in the same direction with respect to the role(s) and importance of protein turnover and adduct persistence in eliciting cytotoxicity.

## Conclusion

The covalent binding of reactive metabolites to cellular proteins has long been associated with the production of acutely cytotoxic effects. The past several years have witnessed much progress toward identifying their reactive metabolites and the specific intracellular proteins that become adducted, often highly selectively, by reactive metabolites from a number of different protoxicants. For the better-studied protoxicants, much is known about the structures and reactivities of their reactive metabolite(s), the structures of the adducts they form on proteins, and the identities of many of their protein targets [6]. For technical reasons it has proven challenging to elucidate which specific residues on a given target protein become modified [46], but progress is being made in this area as well [7].

The challenge has been to use the available information to discover factors that govern the selectivity of adduction (both among and within different proteins), and to discover mechanisms that link protein adduction to cytotoxicity. As of this writing, the TPDB lists 32 compounds whose reactive metabolites present widely varying chemical reactivities and hydrophobicities, and a total of 268 proteins that are modified by reactive in animals or living cells in vitro [9]. However, for only 9 of these 32 compounds, one of which is not cytotoxic, are 15 or more target proteins known, while for 19 other compounds fewer than 7 target proteins are known. Thus, considering the breadth of the phenomenon, the descriptive data are still rather sparse. Little commonality of targets among different protoxins is apparent, and analyses of target proteins per se have failed to illuminate mechanisms linking covalent binding to toxic outcomes.

Because proteins in cells interact specifically and extensively with other proteins, we hypothesized that xenobiotic adduction might disrupt endogenous PPIs and signaling pathways vital to cellular homeostasis and survival. In the current work we found that the human orthologs of 21 common rat or mouse target proteins have 165 direct-interacting partners that participate in a total of 529 PPIs. These 186 proteins are significantly concentrated in several GO categories and KEGG pathways that experimental studies by others have shown to be highly relevant to cell signaling and cell survival. This suggests that compared to direct analysis of target proteins, further bioinformatic analysis of proteins that interact with greater numbers of target proteins may be able to point toward more fruitful areas for generating and testing hypotheses about mechanisms of toxicity.

Finaly, given the chemical and structural diversity of cellular proteins, it seems unlikely that simple principles of chemical reactivity will in themselves play an important role in differentiating target from non-target proteins. As shown by studies with bromophenol, m-hydroxyacetanilide, and mycophenolic acid, not all protein adduction has toxic concequences. It seems equally likely that among adducts generated from toxic compounds, only some but not all will have toxic consequences. It will take time and much more detailed information about protein adduction in living cells to sort this out, and the outcome is likely to point to a number of mechanistic paths from protein modification to cellular impairment or death. In the meantime, one aspect of adduct chemistry that experiments and bioinformatic analysis both suggest may be important is the persistence of the adducted protein in the cell. Unstable adducts that dissociate, and adducted proteins that turnover rapidly, may be less effective at perturbing cellular homeostasis and injuring the cell than more durable adducts.

## Authors' contributions

All authors contributed to the design of the three sub-projects, the interpretation of the results, and the drafting and production of the manuscript. Bioinformatic analyses were conducted by JF. All authors read and approved the final manuscript.

## Supplementary Material

Additional file 1 **Figure S1. Graphic representation of enriched GO Biological Process categories and their hierarchical structure of the 171 target proteins**. Heirarchial tree diagram showing the names and relationships of the GO Biological Process categories in which members of a set of 171 reactive metabolite target proteins are statistically over-represented. Click here for file

Additional file 2 **Figure S2. Graphic representation of enriched GO Molecular Function categories and their hierarchical structure of the 171 target proteins**. Heirarchial tree diagram showing the names and relationships of the GO Molecular Function categories in which members of a set of 171 reactive metabolite target proteins are statistically over-represented. Click here for file

Additional file 3 **Table S1. Human orthologs of 28 common rat/mouse target proteins**. Accession numbers of 28 common rat/mouse target proteins and their human orthologs, and their degree of similarity. Click here for file

Additional file 4 **Table S2. First-partners of 28 common target proteins**. Accession numbers of 28 common rat/mouse target proteins and their human orthologs, and their degree of similarity. Accession numbers and gene symbols of the 165 directly-interacting partners found for 28 common rat and mouse target proteins. Click here for file

Additional file 5 **Table S3. Significantly over-populated GO terms**. GO ID numbers and names of Molecular Function, Biological Process and Cellular Component categories in which members of a set of 171 reactive metabolite target proteins are statistically over-represented. Click here for file
