# Supplementary material for: Bioinformatic analysis of xenobiotic reactive metabolite target proteins and their interacting partners
Source: BMC Chem Biol. 2009 Jun 12;9:5. doi: 10.1186/1472-6769-9-5 (PMC2711050; doi:10.1186/1472-6769-9-5)

**Figure S2.** Graphic representation of enriched GO Molecular Function categories and their hierarchical structure of the 171 target proteins.


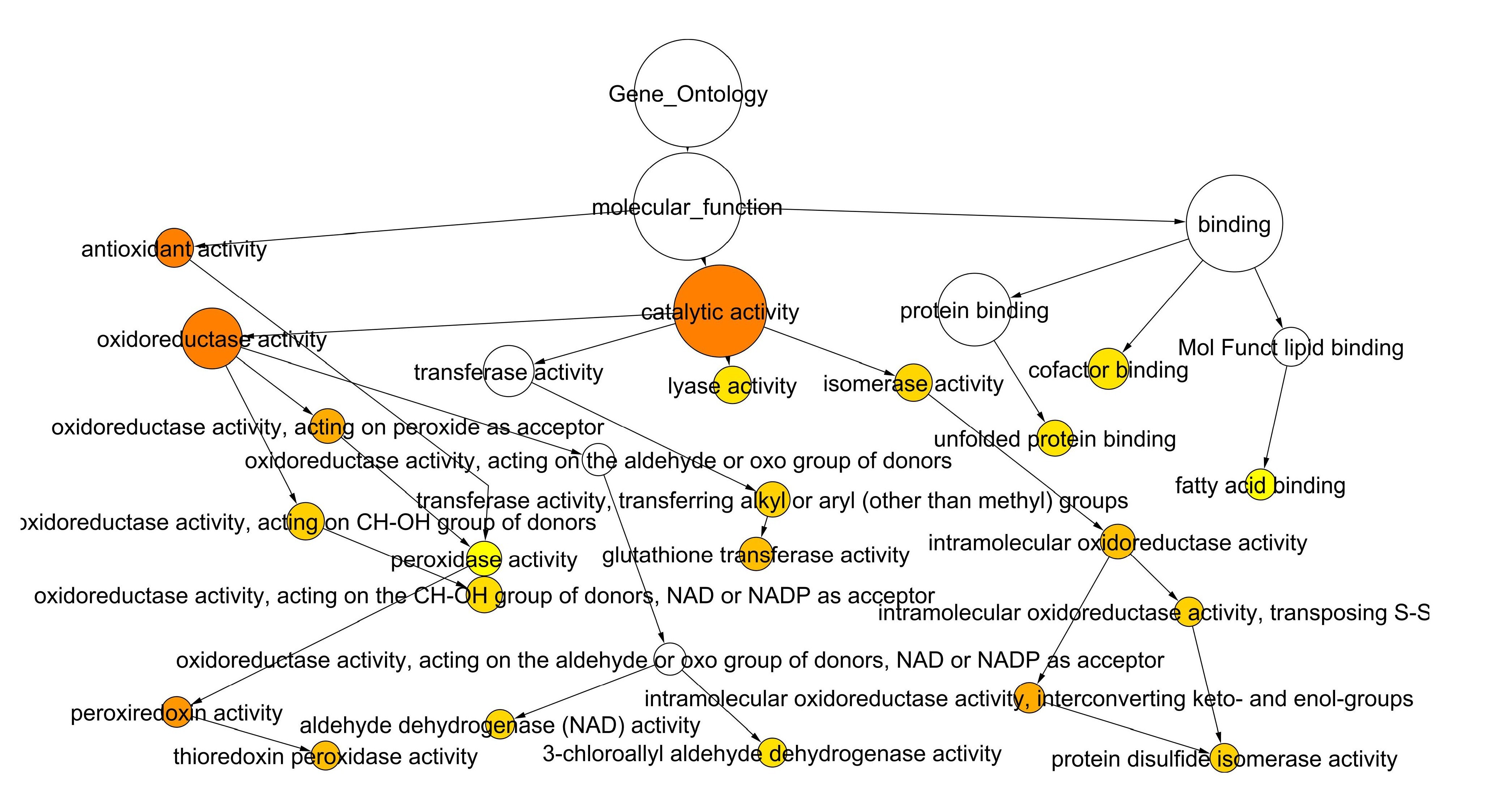

Supplement: Additional file 2 — Figure S2. Graphic representation of enriched GO Molecular Function categories and their hierarchical structure of the 171 target proteins. Heirarchial tree diagram showing the names and relationships of the GO Molecular Function categories in which members of a set of 171 reactive metabolite target proteins are statistically over-represented. [file 1472-6769-9-5-S2.doc]
