# Supplementary material for: Bioinformatic analysis of xenobiotic reactive metabolite target proteins and their interacting partners
Source: BMC Chem Biol. 2009 Jun 12;9:5. doi: 10.1186/1472-6769-9-5 (PMC2711050; doi:10.1186/1472-6769-9-5)
Supplement: Additional file 3 — Table S1. Human orthologs of 28 common rat/mouse target proteins. Accession numbers of 28 common rat/mouse target proteins and their human orthologs, and their degree of similarity. [file 1472-6769-9-5-S3.doc]

**Table S1**. Human orthologs of 28 common rat/mouse target proteins.

| **Rat/mouse**  **protein ID** | **Human**  **protein ID** | **E-value** | **Length of rat or mouse protein** | **Length of human protein** | **Length of aligned rat/mouse protein** | **Length of aligned**  **human protein** | **Percent identity** | **Percent positive** |
| --- | --- | --- | --- | --- | --- | --- | --- | --- |
| P14141 | 13436164 | 4.00E-147 | 260 | 260 | 260 | 260 | 91 | 94 |
| P52555 | 5803013 | 4.00E-132 | 260 | 261 | 260 | 260 | 90 | 94 |
| P47738 | 25777732 | 2.00E-289 | 519 | 517 | 516 | 519 | 95 | 97 |
| P06761 | 86577744 | 0 | 654 | 655 | 654 | 654 | 99 | 99 |
| P04905 | 23065544 | 6.00E-107 | 218 | 218 | 218 | 218 | 79 | 91 |
| Q63836 | 16306550 | 1.00E-257 | 472 | 472 | 472 | 472 | 87 | 93 |
| P02770 | 4502027 | 2.00E-285 | 608 | 609 | 607 | 607 | 73 | 87 |
| P02767 | 4507725 | 4.00E-68 | 147 | 147 | 146 | 146 | 82 | 92 |
| P21107-2 (mouse) | 24119203 | 1.00E-132 | 248 | 248 | 248 | 248 | 99 | 99 |
| P02692 | 4557577 | 3.00E-56 | 127 | 127 | 127 | 127 | 82 | 90 |
| P09103 (mouse) | 20070125 | 3.00E-278 | 509 | 508 | 507 | 509 | 94 | 96 |
| P07687 | 4503583 | 3.00E-239 | 455 | 455 | 455 | 455 | 84 | 92 |
| P27773 | 21361657 | 3.00E-282 | 505 | 505 | 505 | 505 | 93 | 97 |
| Q63081 | 5031973 | 3.00E-248 | 440 | 440 | 440 | 440 | 94 | 97 |
| P11232 | 50592994 | 1.00E-51 | 105 | 105 | 103 | 103 | 90 | 95 |
| P52759 | P52758 | 1.00E-63 | 137 | 137 | 137 | 137 | 86 | 92 |
| P31044 | 4505621 | 3.00E-93 | 187 | 187 | 187 | 187 | 83 | 90 |
| P63018 | 5729877 | 0 | 646 | 646 | 646 | 646 | 99 | 100 |
| P80254 | 4503291 | 7.00E-48 | 118 | 118 | 118 | 118 | 74 | 88 |
| P10868| | 4503909 | 6.00E-125 | 236 | 236 | 235 | 235 | 86 | 95 |
| P04764 | 4503571 | 3.00E-239 | 434 | 434 | 434 | 434 | 94 | 98 |
| P08010 | 23065544 | 2.00E-109 | 218 | 218 | 218 | 218 | 81 | 92 |
| P23457 | 24497585 | 5.00E-134 | 322 | 323 | 322 | 322 | 69 | 83 |
| P07824 | 10947139 | 2.00E-164 | 323 | 322 | 322 | 323 | 87 | 93 |
| Q03248 | 7706509 | 2.00E-197 | 393 | 384 | 384 | 384 | 84 | 91 |
| P41562 | 28178825 | 2.00E-237 | 414 | 414 | 414 | 414 | 96 | 98 |
| P25093 | 4557587 | 5.00E-226 | 419 | 419 | 419 | 419 | 88 | 94 |
| P16617 | 4505763 | 5.00E-235 | 417 | 417 | 417 | 417 | 97 | 98 |
