# Supplementary material for: Bioinformatic analysis of xenobiotic reactive metabolite target proteins and their interacting partners
Source: BMC Chem Biol. 2009 Jun 12;9:5. doi: 10.1186/1472-6769-9-5 (PMC2711050; doi:10.1186/1472-6769-9-5)
Supplement: Additional file 4 — Table S2. First-partners of 28 common target proteins. Accession numbers of 28 common rat/mouse target proteins and their human orthologs, and their degree of similarity. Accession numbers and gene symbols of the 165 directly-interacting partners found for 28 common rat and mouse target proteins. [file 1472-6769-9-5-S4.doc]

**Table S2**. First-partners of 28 common target proteins.

| **Entrez ID** | **Gene symbol** | **Protein name** |
| --- | --- | --- |
| 2 | A2M | Macroglobulin, alpha 2 |
| 58 | ACTA1 | Actin alpha, skeletal muscle 1 |
| 60 | ACTB | Actin beta |
| 156 | ADRBK1 | G protein dependent receptor kinase 2 |
| 229 | ALDOB | Aldolase B, fructose-bisphosphate |
| 259 | AMBP | AMBP protein |
| 328 | APEX1 | Apex nuclease |
| 335 | APOA1 | Apolipoprotein A I |
| 338 | APOB | Apolipoprotein B |
| 348 | APOE | Apolipoprotein E |
| 384 | ARG2 | Arginase II |
| 468 | ATF4 | Activating transcription factor 4 |
| 573 | BAG1 | BAG 1 |
| 780 | DDR1 | Discoidin domain receptor |
| 811 | CALR | Calreticulin |
| 821 | CANX | Calnexin |
| 840 | CASP7 | Caspase 7 |
| 912 | CD1D | CD1d |
| 958 | CD40 | CD40 |
| 1107 | CHD3 | Chromodomain helicase DNA binding protein 3 |
| 1147 | CHUK | IKK alpha |
| 1211 | CLTA | Clathrin light polypeptide A |
| 1264 | CNN1 | Calponin 1 |
| 1277 | COL1A1 | Collagen, type I, alpha 1 |
| 1278 | COL1A2 | Collagen, type I, alpha 2 |
| 1300 | COL10A1 | Collagen, type X, alpha-1 |
| 1400 | CRMP1 | Collapsin response mediator protein 1 |
| 1409 | CRYAA | Crystallin, alpha A |
| 1801 | DPH1 | OVCA1 |
| 1915 | EEF1A1 | Elongation factor 1 alpha 1 |
| 2079 | ERH | ERH |
| 2081 | ERN1 | Endoplasmic reticulum to nucleus signalling 1 |
| 2157 | F8 | Coagulation factor VIII |
| 2203 | FBP1 | Fructose-1,6-bisphosphatase |
| 2217 | FCGRT | Fc fragment of IgG, receptor transporter, alpha |
| 2580 | GAK | Cyclin G associated kinase |
| 2597 | GAPDH | Glyceraldehyde 3 phosphate dehydrogenase |
| 2643 | GCH1 | GTP cyclohydrolase I |
| 2671 | GFER | Hepatopoietin |
| 2806 | GOT2 | Glutamate oxaloacetate transaminase, mitochondrial |
| 2890 | GRIA1 | Glutamate receptor, ionotropic AMPA 1 |
| 2908 | NR3C1 | Glucocorticoid receptor |
| 2920 | CXCL2 | Macrophage inflammatory protein 2 alpha |
| 2947 | GSTM3 | Glutathione S-transferase Mu3 |
| 2995 | GYPC | Glycophorin C |
| 3091 | HIF1A | Hypoxia inducible factor 1 alpha subunit |
| 3105 | HLA-A | HLA-A |
| 3107 | HLA-C | HLA-C |
| 3297 | HSF1 | Heat-shock transcription factor 1 |
| 3301 | DNAJA1 | Heat shock 40 kDa protein 4 |
| 3303 | HSPA1A | Heat shock 70 KD protein 1A |
| 3320 | HSP90AA1 | HSP90A |
| 3329 | HSPD1 | Heat shock 60 KD protein 1 (chaperonin) |
| 3336 | HSPE1 | Heat shock 10 KD protein |
| 3339 | HSPG2 | Perlecan |
| 3359 | HTR3A | 5-hydroxytryptamine (serotonin) receptor 3A |
| 3458 | IFNG | Interferon, gamma |
| 3507 | IGHM | Immunoglobulin mu |
| 3551 | IKBKB | IKK beta |
| 3692 | ITGB4BP | Eukaryotic translation initiation factor 6 |
| 3717 | JAK2 | Janus kinase 2 |
| 3856 | KRT8 | Keratin 8 |
| 3875 | KRT18 | Keratin 18 |
| 3925 | STMN1 | Stathmin 1 |
| 3938 | LCT | Lactase |
| 3949 | LDLR | Low density lipoprotein receptor |
| 4036 | LRP2 | Megalin |
| 4088 | SMAD3 | SMAD3 |
| 4137 | MAPT | Microtubule associated protein tau |
| 4140 | MARK3 | MARK3 |
| 4217 | MAP3K5 | ASK1 |
| 4435 | CITED1 | Melanocyte-specific protein 1 |
| 4653 | MYOC | Myocilin |
| 4689 | NCF4 | Neutrophil cytosolic factor 4, 40kDa |
| 4790 | NFKB1 | NFKB1 |
| 4842 | NOS1 | Nitric oxide synthase 1 |
| 5004 | ORM1 | Alpha-1 acid glycoprotein |
| 5340 | PLG | Plasminogen |
| 5444 | PON1 | Paraoxonase 1 |
| 5465 | PPARA | Peroxisome proliferator activated receptor, alpha |
| 5467 | PPARD | Peroxisome proliferator activated receptor, delta |
| 5468 | PPARG | Peroxisome proliferator activated receptor gamma |
| 5481 | PPID | Peptidyl-prolyl isomerase D |
| 5552 | PRG1 | Proteoglycan 1 |
| 5578 | PRKCA | Protein kinase C alpha |
| 5579 | PRKCB1 | Protein kinase C, beta 1 |
| 5580 | PRKCD | Protein kinase C delta |
| 5582 | PRKCG | Protein kinase C, gamma |
| 5590 | PRKCZ | Protein kinase C, zeta |
| 5594 | MAPK1 | ERK2 |
| 5604 | MAP2K1 | MEK1 |
| 5621 | PRNP | Prion protein |
| 5764 | PTN | Pleiotrophin |
| 5894 | RAF1 | RAF1 |
| 5925 | RB1 | Retinoblastoma 1 |
| 5950 | RBP4 | Retinol binding protein 4 |
| 5966 | REL | C-Rel proto-oncogene protein |
| 5997 | RGS2 | RGS2 |
| 6117 | RPA1 | Replication factor A protein 1 |
| 6273 | S100A2 | S100 calcium binding protein A2 |
| 6275 | S100A4 | S100 calcium binding protein A4 |
| 6513 | SLC2A1 | Solute carrier family,member 1 |
| 6517 | SLC2A4 | Solute carrier family 2 member 4 |
| 6559 | SLC12A3 | Solute carrier family 12 (sodium/chloride transporter), member 3 |
| 6667 | SP1 | Transcription factor Sp1 |
| 6774 | STAT3 | STAT3 |
| 6885 | MAP3K7 | MAP3K7 |
| 6890 | TAP1 | Transporter 1 ATP binding cassette, subfamily B |
| 6927 | TCF1 | Transcription factor 1 |
| 6955 | TRA@ | T cell antigen receptor, alpha |
| 7038 | TG | Thyroglobulin |
| 7111 | TMOD1 | Tropomodulin |
| 7182 | NR2C2 | Nuclear hormone receptor TR4 |
| 7203 | CCT3 | Chaperonin containing T complex polypeptide 1, subunit 1 |
| 7253 | TSHR | Thyroid stimulating hormone receptor |
| 7316 | UBC | Ubiquitin C |
| 7431 | VIM | Vimentin |
| 7450 | VWF | Von Willebrand factor |
| 7529 | YWHAB | 14-3-3 Beta |
| 7532 | YWHAG | 14-3-3 gamma |
| 7534 | YWHAZ | 14-3-3 zeta |
| 7852 | CXCR4 | Chemokine, CXC motif, receptor 4 |
| 8029 | CUBN | Cubilin |
| 8518 | IKBKAP | IKAP |
| 8655 | DYNLL1 | Dynein light chain 1 |
| 8841 | HDAC3 | Histone deacetylase 3 |
| 8974 | P4HA2 | P4HA2 |
| 9020 | MAP3K14 | NIK |
| 9093 | DNAJA3 | TID 1 |
| 9159 | PCSK7 | Prohormone convertase PC7 |
| 9319 | TRIP13 | TRIP13 |
| 9451 | EIF2AK3 | Eukaryotic translation initiation factor 2-alpha kinase 3 |
| 9531 | BAG3 | BCL2 associated athanogene 3 |
| 9532 | BAG2 | BCL2 associated athanogene 2 |
| 9564 | BCAR1 | CRK associated substrate |
| 9869 | SETDB1 | SET domain bifurcated 1 |
| 10006 | ABI1 | ABL interactor 1 |
| 10117 | ENAM | Enamelin |
| 10273 | STUB1 | STIP1 homologous and U box containing protein 1 |
| 10628 | TXNIP | Thioredoxin interacting protein |
| 10808 | HSPH1 | Heat shock protein 105 kDa |
| 10915 | TCERG1 | Transcription elongation regulator 1 |
| 10971 | YWHAQ | 14-3-3 theta |
| 10987 | COPS5 | COP9, subunit 5 |
| 11161 | C14orf1 | C14orf1 protein |
| 23287 | AGTPBP1 | ATP/GTP binding protein 1 |
| 23621 | BACE1 | Beta site App cleaving enzyme |
| 23640 | - | HSPBP1 |
| 29767 | TMOD2 | Tropomodulin 2 |
| 29844 | TFPT | Amida |
| 29979 | UBQLN1 | Ubiquilin 1 |
| 30001 | ERO1L | ERO1-like |
| 51003 | MED31 | Mediator of RNA polymerase II transcription, subunit 31 homolog |
| 54205 | CYCS | Cytochrome C |
| 54431 | DNAJC10 | ERDJ5 |
| 54972 | TMEM132A | GBP protein |
| 55791 | C1orf103 | Receptor-interacting factor 1 |
| 56605 | ERO1LB | ERO1-like beta |
| 56893 | UBQLN4 | Ataxin 1 ubiquitin like interacting protein |
| 57685 | CACHD1 | Hypothetical protein KIAA1573 |
| 64215 | DNAJC1 | HTJ1 |
| 64374 | SIL1 | BiP associated protein |
| 79036 | - | Hypothetical protein MGC2749 |
| 283208 | P4HA3 | Collagen prolyl 4-hydroxylase alpha III subunit |
| 387082 | SUMO4 | SUMO4 |
