# Supplementary material for: Bioinformatic analysis of xenobiotic reactive metabolite target proteins and their interacting partners
Source: BMC Chem Biol. 2009 Jun 12;9:5. doi: 10.1186/1472-6769-9-5 (PMC2711050; doi:10.1186/1472-6769-9-5)
Supplement: Additional file 5 — Table S3. Significantly over-populated GO terms. GO ID numbers and names of Molecular Function, Biological Process and Cellular Component categories in which members of a set of 171 reactive metabolite target proteins are statistically over-represented. [file 1472-6769-9-5-S5.doc]

**Table S3**. Significantly over-popoulated GO terms (Benjamini and Hochberg false discovery rate < 1.0E-03).

| **GO-ID** | **Category Description** | **Corrected p-value** | **Number of target proteins selected** | **Total number of proteins in category** | **Fraction of population as targets** |
| --- | --- | --- | --- | --- | --- |
|  | Molecular Function |  |  |  |  |
| 3824 | catalytic activity | 1.45E-19 | 115 | 5085 | 0.023 |
| 16491 | oxidoreductase activity | 2.76E-12 | 40 | 916 | 0.044 |
| 16209 | antioxidant activity | 2.93E-09 | 11 | 57 | 0.193 |
| 51920 | peroxiredoxin activity | 9.34E-08 | 5 | 6 | 0.833 |
| 16862 | intramolecular oxidoreductase activity, interconverting keto- and enol-groups | 5.71E-07 | 5 | 8 | 0.625 |
| 4601 | peroxidase activity | 5.71E-07 | 8 | 40 | 0.200 |
| 16684 | oxidoreductase activity, acting on peroxide as acceptor | 5.71E-07 | 8 | 40 | 0.200 |
| 4364 | glutathione transferase activity | 3.30E-06 | 7 | 34 | 0.206 |
| 9031 | thioredoxin peroxidase activity | 3.30E-06 | 4 | 5 | 0.800 |
| 16860 | intramolecular oxidoreductase activity | 3.51E-06 | 8 | 52 | 0.154 |
| 16614 | oxidoreductase activity, acting on CH-OH group of donors | 1.34E-05 | 10 | 111 | 0.090 |
| 16864 | intramolecular oxidoreductase activity, transposing S-S bonds | 1.57E-05 | 4 | 7 | 0.571 |
| 3756 | protein disulfide isomerase activity | 1.57E-05 | 4 | 7 | 0.571 |
| 16765 | transferase activity, transferring alkyl or aryl (other than methyl) groups | 1.68E-05 | 8 | 66 | 0.121 |
| 4029 | aldehyde dehydrogenase (NAD) activity | 2.58E-05 | 4 | 8 | 0.500 |
| 16853 | isomerase activity | 2.58E-05 | 10 | 124 | 0.081 |
| 16616 | oxidoreductase activity, acting on the CH-OH group of donors, NAD or NADP as acceptor | 4.09E-05 | 9 | 102 | 0.088 |
| 4028 | 3-chloroallyl aldehyde dehydrogenase activity | 6.65E-05 | 4 | 10 | 0.400 |
| 16829 | lyase activity | 8.82E-05 | 10 | 145 | 0.069 |
| 48037 | cofactor binding | 8.82E-05 | 13 | 255 | 0.051 |
| 51082 | unfolded protein binding | 9.02E-05 | 9 | 115 | 0.078 |
| 5504 | fatty acid binding | 9.42E-04 | 5 | 37 | 0.135 |
|  | ***Biological process*** |  |  |  |  |
| 19752 | carboxylic acid metabolic process | 1.10E-14 | 35 | 524 | 0.067 |
| 6082 | organic acid metabolic process | 1.10E-14 | 35 | 526 | 0.067 |
| 6519 | amino acid and derivative metabolic process | 1.02E-08 | 23 | 362 | 0.064 |
| 9308 | amine metabolic process | 1.53E-08 | 24 | 414 | 0.058 |
| 9056 | catabolic process | 1.53E-08 | 30 | 652 | 0.046 |
| 6807 | nitrogen compound metabolic process | 4.10E-08 | 24 | 439 | 0.055 |
| 8152 | metabolic process | 4.82E-08 | 125 | 7527 | 0.017 |
| 6950 | response to stress | 4.82E-08 | 36 | 978 | 0.037 |
| 44248 | cellular catabolic process | 9.68E-08 | 26 | 548 | 0.047 |
| 6805 | xenobiotic metabolic process | 2.31E-07 | 8 | 32 | 0.250 |
| 9410 | response to xenobiotic stimulus | 2.74E-07 | 8 | 33 | 0.242 |
| 6979 | response to oxidative stress | 4.38E-07 | 12 | 110 | 0.109 |
| 6520 | amino acid metabolic process | 1.28E-06 | 17 | 271 | 0.063 |
| 6457 | protein folding | 1.28E-06 | 15 | 207 | 0.072 |
| 44237 | cellular metabolic process | 1.56E-06 | 112 | 6683 | 0.017 |
| 6732 | coenzyme metabolic process | 1.72E-06 | 14 | 183 | 0.077 |
| 44249 | cellular biosynthetic process | 1.79E-06 | 25 | 605 | 0.041 |
| 51186 | cofactor metabolic process | 3.03E-06 | 15 | 225 | 0.067 |
| 9063 | amino acid catabolic process | 2.56E-05 | 8 | 61 | 0.131 |
| 10038 | response to metal ion | 3.54E-05 | 8 | 64 | 0.125 |
| 32787 | monocarboxylic acid metabolic process | 4.34E-05 | 14 | 243 | 0.058 |
| 10035 | response to inorganic substance | 7.23E-05 | 8 | 71 | 0.113 |
| 9310 | amine catabolic process | 7.70E-05 | 8 | 72 | 0.111 |
| 44270 | nitrogen compound catabolic process | 9.11E-05 | 8 | 74 | 0.108 |
| 6749 | glutathione metabolic process | 1.07E-04 | 5 | 20 | 0.250 |
| 6725 | aromatic compound metabolic process | 1.29E-04 | 10 | 133 | 0.075 |
| 42743 | hydrogen peroxide metabolic process | 1.29E-04 | 4 | 10 | 0.400 |
| 9064 | glutamine family amino acid metabolic process | 1.62E-04 | 6 | 38 | 0.158 |
| 9991 | response to extracellular stimulus | 2.43E-04 | 9 | 114 | 0.079 |
| 6790 | sulfur metabolic process | 2.49E-04 | 8 | 87 | 0.092 |
| 42542 | response to hydrogen peroxide | 2.82E-04 | 5 | 25 | 0.200 |
| 6066 | alcohol metabolic process | 2.90E-04 | 15 | 338 | 0.044 |
| 6525 | arginine metabolic process | 3.49E-04 | 4 | 13 | 0.308 |
| 51 | urea cycle intermediate metabolic process | 4.71E-04 | 4 | 14 | 0.286 |
| 6091 | generation of precursor metabolites and energy | 4.84E-04 | 20 | 591 | 0.034 |
| 9605 | response to external stimulus | 5.70E-04 | 21 | 650 | 0.032 |
| 9065 | glutamine family amino acid catabolic process | 7.72E-04 | 4 | 16 | 0.250 |
| 6096 | glycolysis | 8.41E-04 | 8 | 106 | 0.075 |
|  | ***Celluar component*** |  |  |  |  |
| 44444 | cytoplasmic part | 3.71E-23 | 91 | 2969 | 0.031 |
| 5739 | mitochondrion | 2.31E-16 | 41 | 750 | 0.055 |
| 44424 | intracellular part | 1.16E-14 | 121 | 6666 | 0.018 |
| 43231 | intracellular membrane-bound organelle | 8.43E-13 | 97 | 4687 | 0.021 |
| 43227 | membrane-bound organelle | 8.43E-13 | 97 | 4692 | 0.021 |
| 5622 | intracellular | 1.49E-11 | 122 | 7339 | 0.017 |
| 5788 | endoplasmic reticulum lumen | 4.95E-10 | 8 | 20 | 0.400 |
| 43229 | intracellular organelle | 2.61E-09 | 101 | 5667 | 0.018 |
| 43226 | organelle | 2.66E-09 | 101 | 5677 | 0.018 |
| 5829 | cytosol | 2.95E-09 | 25 | 494 | 0.051 |
| 5783 | endoplasmic reticulum | 9.68E-09 | 25 | 525 | 0.048 |
| 5793 | ER-Golgi intermediate compartment | 2.07E-06 | 6 | 23 | 0.261 |
| 5625 | soluble fraction | 2.56E-06 | 15 | 260 | 0.058 |
| 44432 | endoplasmic reticulum part | 2.53E-05 | 10 | 132 | 0.076 |
| 19866 | organelle inner membrane | 3.87E-05 | 13 | 245 | 0.053 |
| 5615 | extracellular space | 9.09E-05 | 38 | 1663 | 0.023 |
| 5743 | mitochondrial inner membrane | 1.08E-04 | 12 | 232 | 0.052 |
| 31967 | organelle envelope | 1.71E-04 | 16 | 421 | 0.038 |
| 31975 | envelope | 1.73E-04 | 16 | 423 | 0.038 |
| 5740 | mitochondrial envelope | 2.02E-04 | 13 | 293 | 0.044 |
| 44421 | extracellular region part | 2.51E-04 | 38 | 1758 | 0.022 |
| 31966 | mitochondrial membrane | 3.65E-04 | 12 | 269 | 0.045 |
| 44429 | mitochondrial part | 3.65E-04 | 14 | 360 | 0.039 |
